# From checklists to phenotypes: a new era in individualized hcm risk stratification with CMR

**DOI:** 10.1007/s10554-026-03754-1

**Published:** 2026-06-13

**Authors:** Alexander Schulz, Nadine Abanador-Kamper, Grigorios Korosoglou

**Affiliations:** 1 Department of Cardiology and Pneumology, University Medical Center Göttingen, Georg- August University and German Center for Cardiovascular Research (DZHK), Partner Site Lower-Saxony, Göttingen, Germany; 2 Department of Medicine, Cardiovascular Division, Beth Israel Deaconess Medical Center, Harvard Medical School, 330 Brookline Ave, Boston, MA 02215 USA; 3 Heart Center Wuppertal, Faculty of Health/School of Medicine, Witten/Herdecke University, Wuppertal, Germany; 4 Department of Cardiology and Vascular Medicine, GRN Hospital Weinheim, Weinheim, Germany; 5 Cardiac Imaging Center Weinheim, Hector Foundation, Weinheim, Germany

## Abstract

**Figure:**
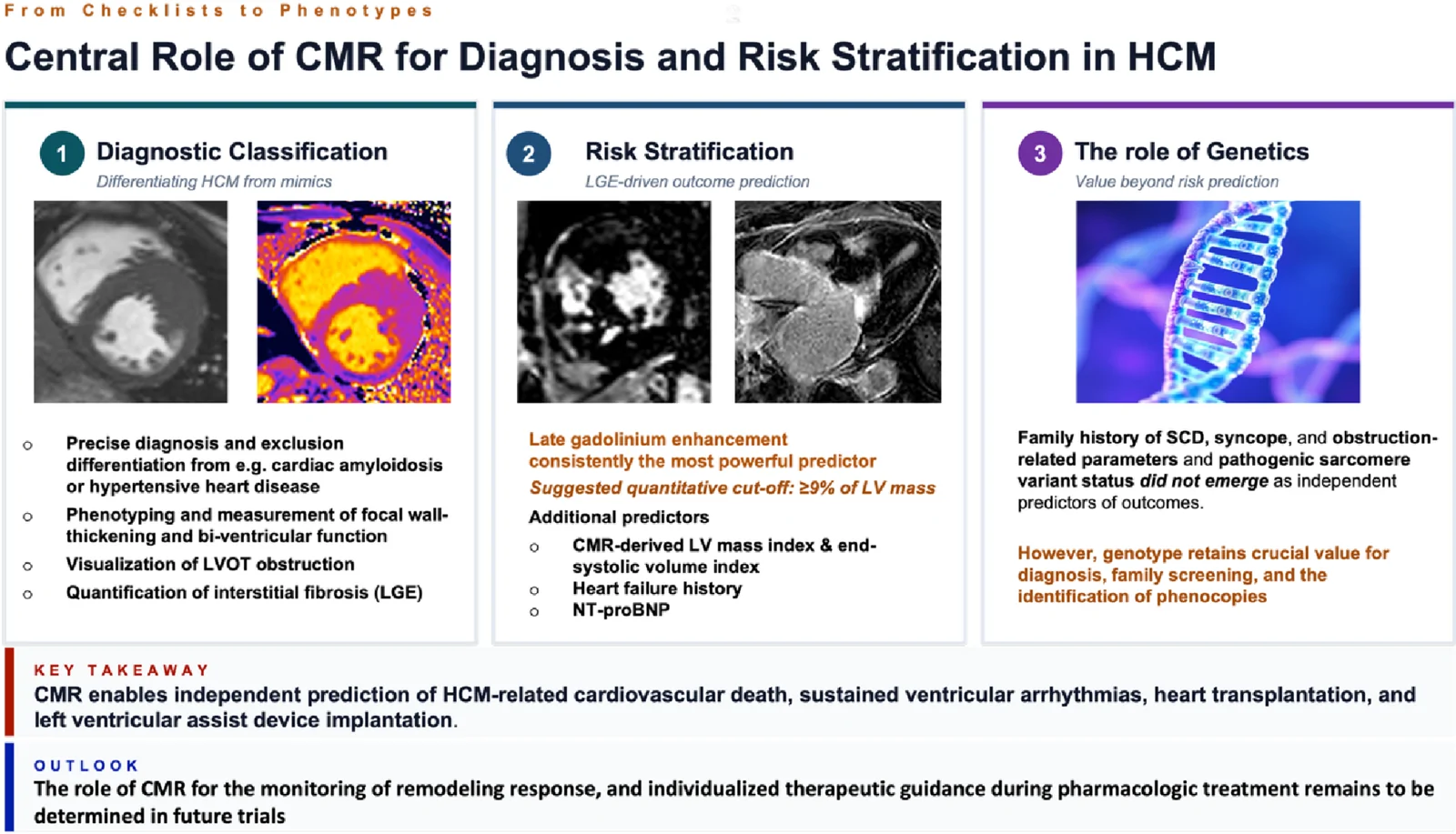

The Central Role of CMR for Diagnosis and Risk Stratification in HCM

Hypertrophic cardiomyopathy (HCM) has long represented one of the greatest challenges in cardiovascular medicine regarding sudden cardiac death (SCD) risk stratification. Although contemporary management strategies have dramatically improved outcomes[1], the disease continues to carry profound uncertainty for both patients and clinicians. Over recent decades, advances in primary and secondary prevention, particularly through broader implementation of implantable cardioverter-defibrillators (ICD), improved heart failure care, and refined septal reduction therapies, have transformed HCM from a disease historically associated with catastrophic outcomes into one with relatively low contemporary mortality in optimally treated cohorts[1, 2]. Yet individualized risk prediction remains imperfect: avoidable sudden deaths still occur, while many patients undergo ICD implantation without ever requiring antiarrhythmic therapy. Current risk stratification models, including international guidelines approaches, have advanced clinical care but remain limited in several respects. Most importantly, they predominantly focus on SCD, even though progressive heart failure, atrial arrhythmias, stroke, and advanced remodeling contribute substantially to long-term morbidity and impaired quality of life in HCM. Traditional risk markers also often rely on simplified or dichotomized assessments that may inadequately capture the remarkable phenotypic heterogeneity of the disease.

Over the last decade, cardiovascular magnetic resonance (CMR) has emerged as a cornerstone in the evaluation of cardiomyopathies, including HCM[3–5]. Contemporary CMR enables comprehensive characterization of hypertrophy distribution, ventricular remodeling, chamber systolic and/or diastolic function and strain, myocardial fibrosis, and subtle structural disease progression. CMR also offers accurate longitudinal assessment of functional and morphological changes over time and increasingly informs procedural planning and therapeutic decision-making[4]. Beyond binary classification of disease presence, CMR increasingly enables quantification of phenotypic disease burden across a continuum[6–8], allowing more refined and individualized characterization of myocardial remodeling and adverse substrate formation.

In this context, the prospective NHLBI Hypertrophic Cardiomyopathy Registry (HCMR) study by Kramer and colleagues represents an important milestone for the field[9]. In this large prospective multicenter registry, 2698 patients from 44 expert centers across North America and Europe underwent standardized clinical assessment, biomarker testing, genotyping, and contrast-enhanced CMR imaging with nearly 7 years of follow-up. Importantly, the investigators moved beyond traditional SCD-focused endpoints, evaluating a broader composite outcome, which encompassed HCM-related cardiovascular death, sustained ventricular arrhythmias, heart transplantation, and left ventricular assist device implantation. The study identified several independent predictors of adverse outcomes, most notably late gadolinium enhancement (LGE) burden, left ventricular (LV) mass index, LV end-systolic volume index, prior heart failure history, and NT-proBNP. CMR-derived markers consistently emerged across both the primary composite endpoint and SCD-related outcomes, with prognostic models achieving excellent discrimination (C-statistics of ~ 0.77).

The importance of this work extends beyond the numerical performance of the models themselves. These findings arise from one of the largest prospectively enrolled and standardized multicenter CMR cohorts in HCM to date, addressing several limitations of prior retrospective studies, which were frequently constrained by referral bias, heterogeneous imaging acquisition and postprocessing approaches. The present study therefore provides particularly robust prospective evidence supporting comprehensive CMR-based phenotyping in HCM, including quantification of myocardial fibrosis.

Several aspects of the findings deserve attention. LV mass index emerged as an independent predictor whereas maximal wall thickness did not. A highly relevant observation, as LV mass represents a comprehensive three-dimensional volumetric assessment of hypertrophic remodeling rather than reliance on a single maximal segment. Similarly, LV end-systolic volume index emerged as an important marker of adverse outcomes, likely reflecting early maladaptive remodeling, and may signal progression toward impaired contractile reserve rather than purely arrhythmic vulnerability. Together with the observation that several traditional risk markers, including family history of SCD, syncope, and obstruction-related parameters, did not emerge as independent predictors of outcomes, these findings suggest that comprehensive CMR phenotyping captures integrated downstream disease expression more effectively than isolated conventional surrogate markers. This should not be interpreted as invalidating established risk markers, particularly given the broader composite endpoint used in the present analysis rather than isolated SCD risk alone, but as a sign to move towards more comprehensive imaging approaches of assessing an HCM heart.

Once more, LGE emerged as the most powerful and consistent predictor of adverse events. What is genuinely new here, however, is the prospective confirmation of this signal within a contemporary multicenter cohort with standardized acquisition and central core-laboratory adjudication, hereby addressing a major limitation of the predominantly retrospective evidence base, on which current guidelines rest. At the same time, the study addresses the so far unresolved question regarding the optimal LGE threshold. In the present analysis, an LGE burden ≥ 9% substantially increased adverse event risk. This aligns closely with recent meta-analytic data suggesting an optimal cutoff around 10%[10], while differing from the > 15% threshold[11] previously propagated in guideline discussions. However, standardization of LGE quantification remains a major challenge. It depends not only on thresholding methodology, but also on contrast agent type, dose and timing of acquisition. Although the current data further strengthens the role of fibrosis quantification in HCM risk assessment, caution is required before universal adoption of any single numerical cutoff. Notably, diffuse interstitial fibrosis assessed by extracellular volume did not emerge as predictive in any of the models, suggesting that dense replacement rather than diffuse fibrosis may represent the more clinically relevant arrhythmogenic and adverse remodeling substrate in HCM.

A particularly intriguing observation concerns the role of genotype. Pathogenic sarcomere variant status was not retained as an independent predictor in any of the models, despite its well-established association with lifetime adverse outcomes. As the authors note, patients with pathogenic variants more frequently exhibit reverse septal curvature morphology, higher LV mass, and more extensive LGE; suggesting that genetic factors may drive the very phenotypic features that ultimately determine risk. In other words, comprehensive imaging-based phenotyping appears to subsume the prognostic information carried by genotype, at least for the long-term outcomes evaluated here. This is consistent with the broader concept that phenotype increasingly trumps genotype for risk stratification in HCM[12], even as genotype retains crucial value for diagnosis, family screening, and identification of phenocopies[5, 13].

Despite these impactful scientific findings, several limitations warrant consideration. Thus, although the cohort size was considerably large, the absolute number of hard events remained relatively modest, particularly for arrhythmic endpoints, potentially limiting broader model development despite sophisticated statistical methodology. Patients with prior ICD implantation were excluded, resulting in a predominantly low- to intermediate-risk cohort with relatively low event rates. While this is likely to enhance applicability to many contemporary outpatient populations, external validation remains essential before broad implementation into clinical risk calculators or guideline recommendations. Moreover, the broad composite endpoint, while clinically meaningful, complicates direct translation into specific decisions such as primary-prevention ICD implantation, or heart failure interventions where clinicians may be concerned of arrhythmic or heart failure risk rather than both. The sudden cardiac death sub-model is in this regard reassuring but was built on only 69 events, and dedicated prospective studies linking the novel CMR risk model to ICD decision-making in contemporary practice remain necessary.

Finally, the study arrives at a particularly important time in the evolution of HCM management. With the approval of mavacamten and emerging data on aficamten, cardiac myosin inhibitors are rapidly reshaping the therapeutic landscape, and comprehensive imaging phenotyping is becoming increasingly relevant not only for prognostication but also for treatment selection, monitoring of remodeling response, and individualized therapeutic guidance. Imaging biomarkers such as quantitative fibrosis burden, ventricular mass, and volumetric remodeling may increasingly evolve from static risk markers into dynamic therapeutic targets. Prospective studies integrating CMR-based phenotyping with novel disease-modifying therapies will be essential to define how these tools should jointly guide clinical decision-making.

Ultimately, the HCMR study strongly reinforces a concept that is increasingly shaping modern HCM care: HCM is not merely a disease of wall thickness, but a complex multidimensional remodeling phenotype requiring comprehensive structural, functional, and tissue characterization. The current study highlights the importance of CMR-derived imaging markers, aiding comprehensive patient phenotyping and individual risk stratification, moving away from a ‘one-fits-all’ approach to precision medicine (Figure). The repeated emergence of CMR-derived markers, now confirmed in the largest prospective contemporary HCM-imaging registries, establishes CMR as no longer merely adjunctive in HCM, but rather the central platform for comprehensive phenotyping and contemporary risk assessment in this disease.

## Data Availability

No datasets were generated or analysed during the current study.

## References

[1] Maron BJ, Ommen SR, Nishimura RA, McKenna WJ, Rakowski H, Sherrid MV, Olivotto I, Braunwald E, Maron MS (2026) Evolution and Transformation of Hypertrophic Cardiomyopathy From a High Risk to a Treatable Low Mortality Disease With Contemporary Clinical Research Strategies. J Am Heart Association 0:e047462. 10.1161/JAHA.125.047462 PMC13323182 41878858

[2] Maron BJ, Rowin EJ, Maron MS (2021) Evolution of risk stratification and sudden death prevention in hypertrophic cardiomyopathy: Twenty years with the implantable cardioverter-defibrillator. Heart Rhythm 18:1012–1023. 10.1016/j.hrthm.2021.01.019 33508516

[3] Rowin Ethan J, Maron Barry J, Maron Martin S (2020) The Hypertrophic Cardiomyopathy Phenotype Viewed Through the Prism of Multimodality Imaging. JACC: Cardiovasc Imaging 13:2002–2016. 10.1016/j.jcmg.2019.09.020 31864978

[4] Rowin EJ, Schulz A (2026) Role of cardiac magnetic resonance imaging in the management of hypertrophic cardiomyopathy. Indian J Thorac Cardiovasc Surg 42:168–180. 10.1007/s12055-025-02039-x 41613491 PMC12847477

[5] Arbelo E, Protonotarios A, Gimeno JR, Arbustini E, Barriales-Villa R, Basso C, Bezzina CR, Biagini E, Blom NA, de Boer RA et al (2023) 2023 ESC Guidelines for the management of cardiomyopathies: Developed by the task force on the management of cardiomyopathies of the European Society of Cardiology (ESC). Eur Heart J 44:3503–3626. 10.1093/eurheartj/ehad194 37622657

[6] Neubauer S, Kolm P, Ho Carolyn Y, Kwong Raymond Y, Desai Milind Y, Dolman Sarahfaye F, Appelbaum E, Desvigne-Nickens P, DiMarco John P, Friedrich Matthias G et al (2019) Distinct Subgroups in Hypertrophic Cardiomyopathy in the NHLBI HCM Registry. JACC 74:2333–2345. 10.1016/j.jacc.2019.08.1057 31699273 PMC6905038

[7] Heydari B, Satriano A, Jerosch-Herold M, Kolm P, Kim D-Y, Cheng K, Choi Yuna L, Antiochos P, White James A, Mahmod M et al (2023) 3-Dimensional Strain Analysis of Hypertrophic Cardiomyopathy. JACC: Cardiovasc Imaging 16:478–491. 10.1016/j.jcmg.2022.10.005 36648040 PMC10802851

[8] Korosoglou G, Ochs M (2023) Spotlight on Myocardial Deformation in Hypertrophic Cardiomyopathy: Putting the Puzzle Together? JACC Cardiovasc Imaging 16:492–494. 10.1016/j.jcmg.2022.11.020 36752433

[9] HCMR-Investigators (2026) Predictors of Long-Term Outcomes in Hypertrophic Cardiomyopathy: The NHLBI HCM Registry. JAMA. 10.1001/jama.2026.5633 PMC13162147 42113540

[10] Kiaos A, Daskalopoulos Georgios N, Kamperidis V, Ziakas A, Efthimiadis G, Karamitsos Theodoros D (2024) Quantitative Late Gadolinium Enhancement Cardiac Magnetic Resonance and Sudden Death in Hypertrophic Cardiomyopathy. JACC Cardiovasc Imaging 17:489–497. 10.1016/j.jcmg.2023.07.005 37632503

[11] Chan RH, Maron BJ, Olivotto I, Pencina MJ, Assenza GE, Haas T, Lesser JR, Gruner C, Crean AM, Rakowski H, Maron MS et al (2014) Prognostic Value of Quantitative Contrast-Enhanced Cardiovascular Magnetic Resonance for the Evaluation of Sudden Death Risk in Patients With Hypertrophic Cardiomyopathy. Circulation 130:484–495. 10.1161/CIRCULATIONAHA.113.007094 25092278

[12] Curran L, de Marvao A, Inglese P, McGurk KA, Schiratti P-R, Clement A, Zheng SL, Li S, Pua CJ, Shah M et al (2023) Genotype-Phenotype Taxonomy of Hypertrophic Cardiomyopathy. Circ Genom Precis Med 16:e004200. 10.1161/CIRCGEN.123.004200 38014537 PMC10729901

[13] Ommen SR, Ho CY, Asif IM, Balaji S, Burke MA, Day SM, Dearani JA, Epps KC, Evanovich L, Ferrari VA, AHA/ACC/AMSSM/ et al (2024) HRS/PACES/SCMR Guideline for the Management of Hypertrophic Cardiomyopathy: A Report of the American Heart Association/American College of Cardiology Joint Committee on Clinical Practice Guidelines. *Circulation*. 2024;149:e1239-e1311. 10.1161/CIR.0000000000001250 38718139

